# Knowledge, attitudes, and practices toward assisted reproductive technology and painless egg retrieval among infertile women in the northwest region of China

**DOI:** 10.3389/fpubh.2025.1614206

**Published:** 2025-10-02

**Authors:** Qiaomei Zhang, Jing Wang, Ting Li, Xiaofei Wang, Juan Geng

**Affiliations:** Department of Anesthesiology, Northwest Women's and Children's Hospital, Xi'An, China

**Keywords:** infertility, assisted reproductive technology, painless egg retrieval, knowledge, attitudes, practice

## Abstract

**Background:**

With the increasing use of assisted reproductive technology (ART) as a solution for infertility, understanding patient knowledge, attitudes, and practices (KAP) is essential. This study aimed to assess the KAP of infertile women in Northwest China regarding ART and painless egg retrieval.

**Methods:**

This cross-sectional study enrolled infertile female population in the Northwest Region of China from April to September.

**Results:**

A total of 403 participants were included, with scores of 12.16 ± 5.63 (range: 0–20) for knowledge, 34.30 ± 4.38 (range: 10–50) for attitudes, and 25.28 ± 4.89 (range: 7–35) for practices. Spearman correlation analysis demonstrated the positive correlations between KAP (*r* = 0.246–0.589, *P* < 0.001). Structural equation modeling (SEM) showed that knowledge and attitudes are key predictors of practices. Notably, knowledge not only directly influenced practice (β = 0.33, *P* < 0.001) but also had an indirect effect through its positive impact on attitudes (β = 0.02, *P* = 0.007).

**Conclusion:**

Infertile women in Northwest China have moderate KAP scores. To improve their adoption of ART, interventions should focus on enhancing their knowledge and improving their attitudes. Tailored support systems, including improved doctor-patient communication and peer support, are particularly vital for women with lower education and less experience with ART.

## Background

Infertility is a multifactorial condition, which refers to the inability to conceive after 1 year of regular, unprotected sexual intercourse (1). Infertility affects around 17.5% of the adult population worldwide, with the highest rates found in the Americas, Europe, and the Western Pacific (2). In China, the overall infertility rate among women of reproductive age is estimated to be 15.5% (3). Additionally, a study conducted in Henan Province, China, found a higher infertility prevalence of 24.58% among women aged 20–49 (4). Egg retrieval is a pivotal component, and painless egg retrieval has gained attention as an innovative solution to reduce the discomfort of traditional procedures (5, 6).

Research has reported that the efficacy of ART and egg retrieval varies considerably among infertile women (7). This variation is influenced not only by clinical factors such as age, underlying medical conditions, and ovarian reserve, but also by the knowledge, attitudes, and practices (KAP) of the women undergoing these treatments (8). Knowledge of ART, risks, success rates, and pain management significantly influences a woman's experience with fertility treatments. An informed patient can make decisions aligned with the goals, reduce anxiety, and improve treatment adherence. Positive attitudes toward ART, including acceptance of painless egg retrieval, enhance psychological outcomes and treatment satisfaction. As for practices, women undergoing ART typically engage in medical interventions such as ovarian stimulation, hormone injections, and egg retrieval (8). To optimize success, maintaining a healthy lifestyle, adhering to medical advice on hormone therapies, and managing stress through psychological support are essential (9). Additionally, clear communication with healthcare providers about pain management preferences, including painless egg retrieval, ensures personalized treatment plans.

The KAP study has been commonly used to provide valuable insights into gaps in understanding, positive attitudes and behaviors in a certain health issue (10). Despite the increasing prevalence of infertility, limited KAP evidence has been available regarding painless egg retrieval. A study in China explored women undergoing *in vitro* fertilization and embryo transfer (IVF-ET), who had inadequate knowledge and negative attitudes, but proactive engagement toward the procedure (11). However, no comprehensive KAP study has been conducted on the broader range of assisted reproductive technologies (ART), including painless egg retrieval, or the interrelationships between KAP.

This study aimed to investigate the KAP of infertile women in the Northwest Region of China regarding ART and painless egg retrieval. The following hypotheses were formulated: (H1) Infertile women's knowledge positively influences their attitudes; (H2) Infertile women's knowledge positively impacts their practices; (H3) Infertile women's attitudes positively affect their practices. By addressing this gap, the study sought to provide a deeper understanding of how these factors shape women's experiences with ART, guide health interventions, and improve patient care.

## Methods

### Study design and subjects

This cross-sectional study enrolled infertile female population in the Northwest Region of China from April to September. Inclusion criteria: (1) females aged 18 years or older with a desire for fertility treatment; (2) complete medical records available for review; (3) Informed consent and willingness to participate in the survey. Exclusion criteria: (1) presence of endometriosis, adenomyosis, uterine malformations, or other relevant gynecological conditions; (2) presence of mental disorders that may impede participation in the questionnaire survey. This study was approved by the Clinical Research Management Committee of Northwest Women's and Children's Hospital (No. 2024-041), and informed consent was obtained from the study participants.

### Questionnaire introduction

A self-designed questionnaire was developed based on existing guidelines and literature (12, 13). The final questionnaire, in Chinese, covered four areas: demographic data, knowledge, attitudes, and practices. The knowledge dimension consisted of 10 questions scored as follows: “Very familiar” for 2 points, “Heard of it” for 1 point, and “Not clear” for 0 points. The total score in the dimension ranged from 0 to 20 points. The attitude dimension included 10 questions on a five-point Likert scale, from “Strongly disagree” (1 point) to “Strongly agree” (5 points). Reverse scoring was applied for items 2, 5, 8, 9, and 10. The total score in the dimension ranged from 10 to 50 points. The practice dimension comprised 7 questions on a five-point Likert scale, from “Always” (5 points) to “Never” (1 point). The total score in the dimension ranged from 7 to 35 points. Overall KAP scores were categorized using Bloom's cut-off values: good (80%−100%), moderate (60%−79%), and poor (< 60%) (14).

### Questionnaire distribution and quality control

The survey was distributed via the WeChat-based Wenjuanxing mini-program, accessible through a QR code. To ensure data integrity, only one submission per IP address was allowed, and all questions were mandatory. The research team reviewed completed questionnaires for accuracy, internal consistency, and logical coherence. Responses were excluded if they met any of the following criteria: completion time under 90 s, inconsistent answers, incorrect responses to trap questions, multiple selections, or uniform answers across all KAP items.

A pre-test was conducted among 32 participants with valid responses. The overall Cronbach's α coefficient was 0.891, with 0.935 for knowledge, 0.822 for attitude, and 0.895 for practice, indicating strong internal consistency. In addition to reliability, content validity was ensured by an expert panel of reproductive medicine and nursing specialists who evaluated item relevance and clarity. Face validity was confirmed during the pre-test by collecting participant feedback. Construct validity was assessed using confirmatory factor analysis (CFA), and the results demonstrated good model fit. Detailed factor loadings and fit indices are provided in Supplementary File 1.

### Sample size calculation

The calculation of sample size was based on the following formula employed in the cross-sectional study (15):


(1) $$\begin{array}{l} n={\left(\frac{Z_{1-\frac{\alpha }{2}}}{\delta }\right)}^{2}\times p\times \left(1-p\right) \end{array}$$

where *n* denoted the sample size. Besides, *p* value was assumed to be 0.5 to achieve the maximum sample size. *a* refers to the type I error, which was set to 0.05 in this case. Subsequently, $Z_{1-\frac{\alpha }{2}}$ was yielded 1.96. δ represents the effect sizes between groups, which was determined as 0.05, and at least 384 participants should be required.

### Statistical methods

Statistical analyses were conducted using SPSS 26.0 and AMOS 24.0 (IBM, Armonk, NY, USA). Normality of continuous data was assessed using the Kolmogorov-Smirnov test. For normally distributed data, results are presented as means ± standard deviations (SD), with comparisons made using Student's *t*-test or ANOVA. Non-normally distributed variables are presented as medians (ranges), with comparisons using the Wilcoxon–Mann–Whitney *U*-test or Kruskal–Wallis test. Categorical variables are expressed as frequencies (*n*, %). Spearman's correlation assessed relationships between KAP scores. Multivariate linear regression identified factors influencing practice scores. Structural equation modeling (SEM), based on the KAP framework, examined whether attitudes mediate the relationship between knowledge and practice. Direct and indirect effect sizes were computed and compared. Model fit was evaluated using indices including root mean square error of approximation (RMSEA), standardized root mean square residual (SRMR), Tucker-Lewis index (TLI), and comparative fit index (CFI). A two-tailed *P*-value < 0.05 was considered statistically significant.

## Results

### Baseline participant information

A total of 512 samples were collected, with 109 samples excluded due to the following reasons: one sample had a response time < 90 s, two samples answered trap questions incorrectly, 49 samples had multiple selections for question 7 in the knowledge section, and 55 samples had multiple selections for question 4 in the attitude section. The final dataset consisted of 403 valid participants. The majority of participants were aged ≥33 years (52.61%), urban residents (56.33%), employed (56.33%), earned a monthly income < 5,000 Chinese Yuan (59.55%), and were married (97.77%). Most participants (80.89%) had only social health insurance, including employee health insurance, “New Rural Cooperative,” and “Urban Resident Insurance.” Among the participants, 44.17% had undergone IVF, and 34.99% planned to undergo IVF in the near future. Additionally, 38.46% had undergone painless egg retrieval, and 36.23% planned to do so in the near future (Table 1).

**Table 1:** Baseline characteristics of infertile women in the Northwest Region of China.

***N* = 403**	***N* (%)**	**Knowledge score**		**Attitude score**		**Practice score**	
		**Mean** **±SD**	***P***	**Mean** **±SD**	***P***	**Mean** **±SD**	***P***
**Total score**		12.16 ± 5.63		34.30 ± 4.38		25.28 ± 4.89	
**Age (year)**	33.15 ± 4.92		1.000		0.108		0.914
< 33	191 (47.39)	12.05 ± 5.59		33.98 ± 4.37		25.26 ± 4.71	
≥33	212 (52.61)	12.25 ± 5.66		34.58 ± 4.37		25.29 ± 5.04	
**Body mass index (kg/m** ^**2**^ **)**	21.32 ± 2.38						
**Residence**			0.128		0.200		0.973
Rural/Suburban	176 (43.67)	11.82 ± 5.44		33.97 ± 4.15		25.39 ± 4.52	
Urban	227 (56.33)	12.41 ± 5.76		34.55 ± 4.53		25.19 ± 5.15	
**Education level**			**0.047**		**0.003**		0.126
High school/technical secondary school or below	145 (35.98)	11.48 ± 5.62		33.47 ± 4.01		24.86 ± 4.73	
Associate degree	106 (26.3)	12.06 ± 5.58		34.06 ± 4.31		25.30 ± 4.87	
Bachelor's degree or above	152 (37.72)	12.86 ± 5.61		35.25 ± 4.60		25.67 ± 5.02	
**Employment status**			0.892		0.062		0.132
Employed	227 (56.33)	12.06 ± 5.68		34.66 ± 4.45		24.87 ± 5.03	
Unemployed	176 (43.67)	12.28 ± 5.56		33.82 ± 4.25		25.80 ± 4.64	
**Monthly income (Chinese Yuan)**			0.875		0.268		0.970
< 5,000	240 (59.55)	12.07 ± 5.54		34.04 ± 4.37		25.32 ± 4.49	
5,000-10,000	120 (29.78)	12.39 ± 5.83		34.52 ± 4.52		25.16 ± 5.66	
>10,000	43 (10.67)	11.95 ± 5.61		35.11 ± 3.94		25.34 ± 4.76	
**Marital status**			0.063		0.179		**0.013**
Single	9 (2.23)	8.444 ± 6.91		32.33 ± 4.06		20.77 ± 6.86	
Married	394 (97.77)	12.24 ± 5.57		34.34 ± 4.38		25.38 ± 4.79	
**Health insurance type**			0.354		0.348		0.718
Only social health insurance (e.g., employee health insurance, “New Rural Cooperative,” “Urban Resident Insurance,” etc.)	326 (80.89)	12.11 ± 5.62		34.32 ± 4.32		25.28 ± 4.81	
Only commercial health insurance	5 (1.24)	9.40 ± 7.79		33.40 ± 4.87		22.60 ± 9.39	
Both social and commercial health insurance	36 (8.93)	13.30 ± 5.97		35.05 ± 4.89		24.86 ± 5.37	
No insurance	36 (8.93)	11.75 ± 5.01		33.44 ± 4.30		26.08 ± 4.26	
**Assisted reproductive technology**			**< 0.001**		**0.002**		**< 0.001**
Have undergone *in vitro* fertilization (IVF)	9 (2.23)	15.33 ± 5.43		36 ± 5.65		27.77 ± 5.23	
Have undergone IVF	178 (44.17)	14.31 ± 5.04		34.62 ± 4.58		27.23 ± 3.83	
Plan to undergo IVF in the near future	141 (34.99)	12.13 ± 4.97		34.60 ± 4.45		25.85 ± 3.96	
No relevant plans in the near future	75 (18.61)	6.706 ± 4.32		32.74 ± 3.11		19.26 ± 3.80	
**Painless egg retrieval surgery**			**< 0.001**		**0.003**		**< 0.001**
Have undergone painless egg retrieval	155 (38.46)	14.87 ± 5.09		34.90 ± 4.70		27.49 ± 3.98	
Plan to undergo painless egg retrieval in the near future	146 (36.23)	12.42 ± 4.83		34.45 ± 4.44		26.32 ± 3.75	
No relevant plans in the near future	102 (25.31)	7.647 ± 4.57		33.16 ± 3.52		20.42 ± 4.19	

Bold values indicate statistical significance at *P* < 0.05.

### Knowledge dimension

The average knowledge score was 12.16 ± 5.63. Knowledge scores varied significantly by education level (*P* = 0.047), experience with ART (*P* < 0.001), and experience with painless egg retrieval (*P* < 0.001) (Table 1). Familiarity with knowledge items ranged from 17.12% to 39.70%. The highest familiarity (39.70%) was with the statement that ART helps infertility patients achieve reproductive goals (K1), and with the definition of painless egg retrieval (K6). The lowest familiarity (17.12%) was with the potential side effects and risks of painless egg retrieval, such as ovarian hyperstimulation syndrome and ovarian infection (K11). Furthermore, only 19.60% were familiar with the item that pain during egg retrieval results from the needle passing through the vaginal fornix, and sometimes the cervix or uterus (K8) (Table 2).

**Table 2:** Distribution of knowledge, attitude, and practice (KAP) scores among participants.

**Knowledge**			**Very familiar *n* (%)**	**Heard of it *n* (%)**	**Not clear *n* (%)**
1. Assisted reproductive technology is a treatment method that helps infertility patients achieve their reproductive desires.			160 (39.7)	220 (54.59)	23 (5.71)
2. Assisted reproductive technology includes artificial insemination and *in vitro* fertilization-embryo transfer (commonly known as IVF), as well as various derived technologies.			151 (37.47)	233 (57.82)	19 (4.71)
3. During *in vitro* fertilization, the eggs and sperm are fertilized outside the body, and the resulting embryos are then transferred into the uterus to promote pregnancy.			145 (35.98)	234 (58.06)	24 (5.96)
4. IVF refers to the technique where eggs are retrieved from a woman's body, cultured in a dish, combined with processed sperm, and once fertilization occurs, the embryo is further cultured until early-stage embryos are formed, at which point they are transferred into the uterus to implant and develop into a fetus until delivery.			151 (37.47)	226 (56.08)	26 (6.45)
5. The capital of China is Shanghai.			106 (26.3)	238 (59.06)	59 (14.64)
6. Painless egg retrieval refers to the process where, under intravenous general anesthesia, a thin needle is used to retrieve mature eggs from the ovaries with ultrasound guidance, for subsequent use in *in vitro* fertilization.			160 (39.7)	220 (54.59)	23 (5.71)
7. Painless egg retrieval surgery can obtain multiple mature eggs, providing more choices for the *in vitro* fertilization process, thereby improving the chances of successful pregnancy. The entire process usually takes about 15-20 minutes.			90 (22.33)	223 (55.33)	90 (22.33)
8. The pain during egg retrieval mainly occurs because the egg retrieval needle must pass through the vaginal fornix, and in some cases, through the cervix or even the uterus.			79 (19.6)	201 (49.88)	123 (30.52)
9. Painless egg retrieval uses a fast-acting, short-duration, and low-side-effect anesthetic drug, which can significantly reduce pain and anxiety during the procedure.			89 (22.08)	226 (56.08)	88 (21.84)
10. Patients with heart disease and severe arrhythmias cannot undergo anesthesia and are not suitable for ART in the short term.			94 (23.33)	210 (52.11)	99 (24.57)
11. I know that painless egg retrieval surgery may lead to side effects and risks, such as ovarian hyperstimulation syndrome and ovarian infection.			69 (17.12)	218 (54.09)	116 (28.78)
**Attitude**	**Strongly agree** ***n*** **(%)**	**Agree** ***n*** **(%)**	**Neutral** ***n*** **(%)**	**Disagree** ***n*** **(%)**	**Strongly disagree** ***n*** **(%)**
1. I believe assisted reproductive technology is a reliable method for helping infertile patients achieve their reproductive desires, bringing new hope for many couples to have children. (*P*)	144 (35.73)	230 (57.07)	26 (6.45)	1 (0.25)	2 (0.5)
2. I oppose infertile women using assisted reproductive technology, believing it is an improper intervention in the natural process. (*N*)	17 (4.22)	51 (12.66)	64 (15.88)	203 (50.37)	68 (16.87)
3. I believe anesthesia does not have an adverse effect on egg quality or later pregnancy. (*P*)	35 (8.68)	175 (43.42)	148 (36.72)	42 (10.42)	3 (0.74)
4. I believe painless egg retrieval surgery provides more choices and opportunities for infertile women, helping them achieve their reproductive desires. (*P*)	114 (28.29)	235 (58.31)	50 (12.41)	3 (0.74)	1 (0.25)
5. I believe painless egg retrieval surgery is an unnecessary invasive procedure that may cause long-term harm to the body. (*N*)	12 (2.98)	61 (15.14)	146 (36.23)	161 (39.95)	23 (5.71)
6. I have full trust in the abilities and professionalism of doctors. (*P*)	137 (34)	227 (56.33)	36 (8.93)	/	3 (0.74)
7. I believe society should provide more support and resources for infertile women, so they can more easily access assisted reproductive technologies and painless egg retrieval surgeries. (*P*)	159 (39.45)	197 (48.88)	44 (10.92)	2 (0.5)	1 (0.25)
8. I am very concerned about the side effects of painless egg retrieval in ART. (*N*)	24 (5.96)	129 (32.01)	194 (48.14)	43 (10.67)	13 (3.23)
9. I believe assisted reproductive technology contradicts the family planning and eugenics policies. (*N*)	44 (10.92)	111 (27.54)	80 (19.85)	134 (33.25)	34 (8.44)
10. I believe assisted reproductive technology contradicts social ethics. (*N*)	16 (3.97)	54 (13.4)	89 (22.08)	198 (49.13)	46 (11.41)
**Practice**	**Always** ***n*** **(%)**	**Often** ***n*** **(%)**	**Sometimes** ***n*** **(%)**	**Rarely** ***n*** **(%)**	**Never** ***n*** **(%)**
1. Are you willing to try assisted reproductive technology (e.g., *in vitro* fertilization or IVF) to achieve your reproductive desires? (*P*)	102 (25.31)	231 (57.32)	57 (14.14)	11 (2.73)	2 (0.5)
2. Are you willing to undergo painless egg retrieval surgery as part of the ART process? (*P*)	87 (21.59)	233 (57.82)	71 (17.62)	12 (2.98)	
3. Are you willing to participate in free medical consultations and educational activities to learn more about assisted reproductive technology and painless egg retrieval surgery? (*P*)	73 (18.11)	227 (56.33)	92 (22.83)	10 (2.48)	1 (0.25)
4. Are you willing to discuss or share information and experiences about assisted reproductive technology and painless egg retrieval surgery with other infertile women? (*P*)	74 (18.36)	231 (57.32)	84 (20.84)	13 (3.23)	1 (0.25)
5. Have you actively sought to learn more about assisted reproductive technology (e.g., *in vitro* fertilization) and painless egg retrieval surgery? (*P*)	39 (9.68)	111 (27.54)	169 (41.94)	64 (15.88)	20 (4.96)
6. Have you communicated and consulted with a doctor or professional about assisted reproductive technology and painless egg retrieval surgery? (*P*)	30 (7.44)	85 (21.09)	167 (41.44)	90 (22.33)	31 (7.69)
7. Have you discussed with your family or partner the possibility of undergoing assisted reproductive technology or painless egg retrieval surgery? (*P*)	45 (11.17)	128 (31.76)	144 (35.73)	62 (15.38)	24 (5.96)

*P* means positive; *N* means negative.

### Attitude dimension

The mean attitude score was 34.30 ± 4.38. Attitude scores varied significantly by education level (*P* = 0.003), experience with ART (*P* = 0.002), and experience with painless egg retrieval (*P* = 0.003) (Table 1). Positive responses (i.e., “Strongly agree” and “Agree” for positive attitude items, and “Strongly disagree” and “Disagree” for negative attitude items) ranged from 13.90% to 92.80%. The highest agreement (92.80%) was with the statement that ART is a reliable method for helping infertile patients achieve reproductive goals, offering hope to many couples (A1). The lowest agreement (13.90%) was with the statement that painless egg retrieval has no concerning side effects (A8). Additionally, a limited proportion (41.69%) expressed a positive attitude toward ART not conflicting with family planning and eugenics policies (A9) (Table 2).

### Practice dimension

The average practice score was 25.28 ± 4.89. Practice scores differed significantly by marital status (*P* = 0.013), experience with ART (P < 0.001), and experience with painless egg retrieval (*P* < 0.001) (Table 1). Adherence to practice items (“Always” and “Often”) ranged from 28.53% to 82.63%. The highest adherence (82.63%) was to the willingness to try ART (e.g., IVF) to achieve reproductive goals (P1). The lowest adherence (28.53%) was to communicating and consulting with a doctor or professional about ART and painless egg retrieval (P6). Furthermore, only 37.22% of participants actively sought more information about ART and painless egg retrieval (P5) (Table 2).

### Spearman correlation

Spearman's correlation analysis revealed significant positive associations between knowledge and attitude (*r* = 0.246, *P* < 0.001), knowledge and practice (r=0.589, P < 0.001), and attitude and practice (*r* = 0.301, *P* < 0.001) (Supplementary Table S1).

### Linear regression analysis

Multivariate regression analysis indicated that both knowledge (β = 0.31, 95% CI: 0.23, 0.38, *P* < 0.001) and attitude (β = 0.15, 95% CI: 0.07, 0.23, *P* < 0.001) were positively associated with practice. In contrast, the absence of near-term plans for ART (β = −3.83, 95% CI: −6.53, −1.14, *P* = 0.005) and painless egg retrieval (β = −2.01, 95% CI: −3.59, −0.43, *P* = 0.013) negatively influenced practice (Table 3).

**Table 3:** Multivariate linear regression analysis of factors influencing practice scores.

**Practice**	**Multivariate analysis**	
	***β*** **(95% CI)**	***P***
**Knowledge score**	0.31 (0.23, 0.38)	**< 0.001**
**Attitude score**	0.15 (0.07, 0.23)	**< 0.001**
**Age (year)**		
< 33		
≥33	0.03 (−0.65, 0.73)	0.912
**Body mass index (kg/m** ^**2**^ **)**	−0.07 (−0.21, 0.06)	0.303
**Residence**		
Rural/Suburban		
Urban	−0.19 (−1.00, 0.61)	0.640
**Education level**		
High school/technical secondary school or below		
Associate degree	0.475 (−0.47, 1.42)	0.327
Bachelor's degree or above	0.871 (−0.16, 1.91)	0.100
**Employment status**		
Employed		
Unemployed	0.721 (−0.09, 1.53)	0.082
**Monthly income (Chinese Yuan)**		
< 5,000		
5,000–10,000	0.107 (−0.70, 0.92)	0.795
>10,000	0.424 (−0.80, 1.65)	0.497
**Marital status**		
Single		
Married	0.340 (−2.04, 2.74)	0.775
**Health insurance type**		
Only social health insurance (e.g., employee health insurance, “New Rural Cooperative,” “Urban Resident Insurance,” etc.)		
Only commercial health insurance	−0.69 (−3.80, 2.41)	0.662
Both social and commercial health insurance	−0.60 (−1.87, 0.66)	0.349
No insurance	0.45 (−0.79, 1.70)	0.477
**Assisted reproductive technology**		
Have undergone *in vitro* fertilization (IVF)		
Have undergone IVF	−0.57 (−2.93, 1.77)	0.629
Plan to undergo IVF in the near future	−1.03 (−3.45, 1.38)	0.402
No relevant plans in the near future	−3.83 −6.53, −1.14)	**0.005**
**Painless egg retrieval surgery**		
Have undergone painless egg retrieval		
Plan to undergo painless egg retrieval in the near future	−0.00 (−1.05, 1.04)	0.992
No relevant plans in the near future	−2.01 (−3.59, −0.43)	**0.013**

### SEM analysis

**Figure 1:**
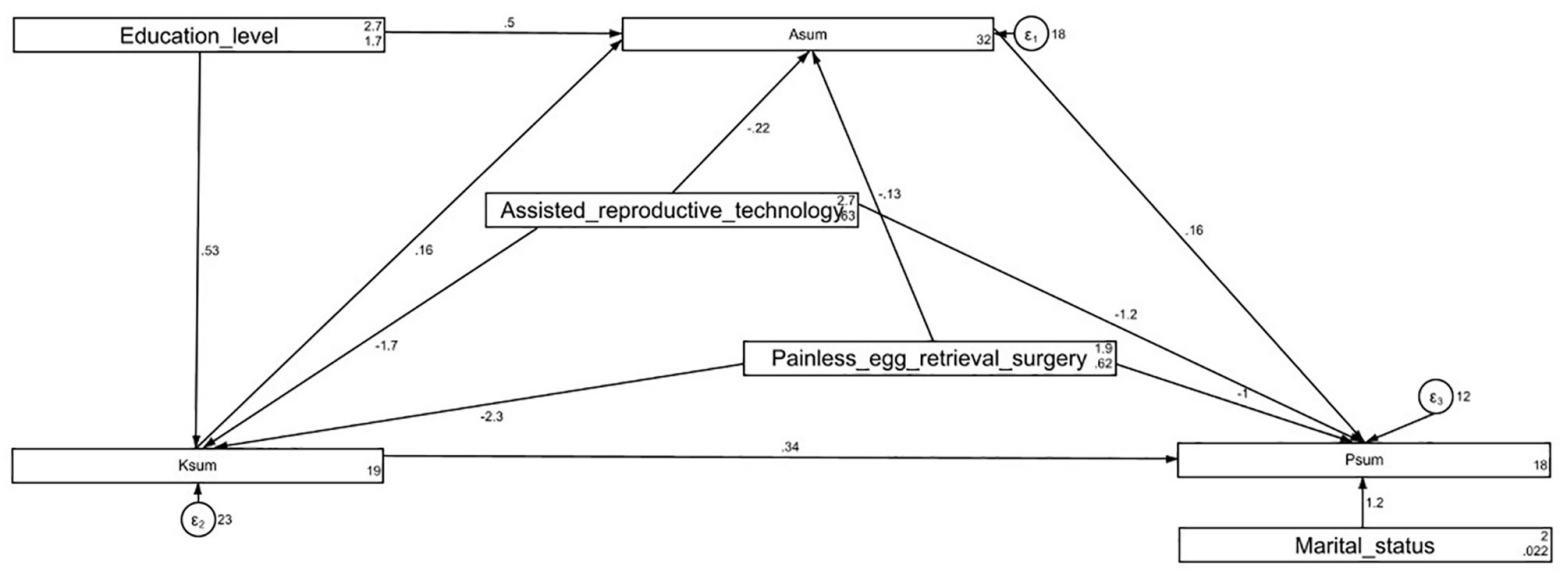
Structural equation model (SEM) model analysis results of KAP scores. All variables are observed variables. Direction of causality is indicated by single-headed arrows. The standardized path coefficients are presented alongside the arrows.

SEM analysis demonstrated a good model fit (RMSEA < 0.001, SRMR=0.009, TLI = 1.025, CFI = 1.000) (Supplementary Table S2). The analysis showed direct effects of education level (β = 0.52, 95% CI: 0.16, 0.88, *P* = 0.004), acceptance of ART (β = −1.65, 95% CI: −2.59,−0.71, *P* = 0.001), and acceptance of painless egg retrieval (β = −2.25, 95% CI: −3.19, −1.30, *P* < 0.001) on knowledge. Knowledge (β = 0.15, 95% CI: 0.07, 0.24, *P* < 0.001) and education level (β = 0.49, 95% CI: 0.18, 0.81, *P* = 0.002) directly impacted attitudes. Additionally, knowledge (β = 0.33, 95% CI: 0.26, 0.40, *P* < 0.001), attitude (β = 0.16, 95% CI: 0.08, 0.24, *P* < 0.001), acceptance of ART (β = −1.23, 95% CI: −1.94, −0.53, *P* = 0.001), and acceptance of painless egg retrieval (β = −1.03, 95% CI: −1.74, −0.32, *P* = 0.005) had direct effects on practice. Furthermore, knowledge (β = 0.02, 95% CI: 0.00, 0.04, *P* = 0.007), education level (β = 0.27, 95% CI: 0.12, 0.42, *P* < 0.001), acceptance of ART (β = −0.63, 95% CI: −1.02, −0.24, *P* = 0.001), and acceptance of painless egg retrieval (β = −0.83, 95% CI: −1.23, −0.43, *P* < 0.001) were indirectly associated with practice (Table 4, Figure 1). The structural equation model (SEM) was constructed based on the KAP theoretical framework, in which knowledge was hypothesized to influence attitudes, and both knowledge and attitudes were expected to affect practices. This pathway (knowledge → attitude → practice) reflects the assumption that greater understanding of ART and painless egg retrieval promotes more positive attitudes, which in turn facilitate corresponding health behaviors. Education level and prior acceptance of ART or painless egg retrieval were included as exogenous variables to capture demographic and experiential influences on knowledge, attitudes, and practices. The path diagram is provided in Supplementary File 1.

**Table 4:** Structural equation modeling (SEM) analysis of direct and indirect effects among KAP scores.

**Model paths**	**Total effects**		**Direct Effect**		**Indirect effect**	
	***β*** **(95% CI)**	***P***	***β*** **(95% CI)**	***P***	***β*** **(95% CI)**	***P***
**Knowledge section**						
Education level → Knowledge	0.52 (0.16, 0.88)	**0.004**	0.52 (0.16, 0.88)	**0.004**	–	–
Acceptance of assisted reproductive technology → Knowledge	−1.65 (−2.59, −0.71)	**0.001**	−1.65 (−2.59, −0.71)	**0.001**	–	–
Acceptance of painless egg retrieval surgery → Knowledge	−2.25 (−3.19, −1.30)	**< 0.001**	−2.25 (−3.19, −1.30)	**< 0.001**	–	–
**Attitude section**						
Knowledge → Attitude	0.15 (0.07, 0.24)	**< 0.001**	0.15 (0.07, 0.24)	**< 0.001**	–	–
Education level → Attitude	0.58 (0.26, 0.90)	**< 0.001**	0.49 (0.18, 0.81)	**0.002**	0.08 (0.01, 0.15)	**0.024**
Acceptance of assisted reproductive technology → Attitude	−0.48 (−1.32, 0.35)	0.260	−0.21 (−1.05, 0.61)	0.610	−0.26 (−0.47, −0.05)	**0.012**
Acceptance of painless egg retrieval surgery → Attitude	−0.48 (−1.32, 0.35)	0.259	−0.12 (−0.97, 0.72)	0.770	−0.35 (−0.60, −0.11)	**0.004**
**Practice section**						
Attitude → Practice	0.16 (0.08, 0.24)	**< 0.001**	0.16 (0.08, 0.24)	**< 0.001**	–	–
Knowledge → Practice	0.36 (0.28, 0.43)	**< 0.001**	0.33 (0.26, 0.40)	**< 0.001**	0.02 (0.00, 0.04)	**0.007**
Education level → Practice	0.27 (0.12, 0.42)	**< 0.001**	–	–	0.27 (0.12, 0.42)	**< 0.001**
Marital status → Practice	1.191 (−1.16, 3.54)	0.321	1.191 (−1.16, 3.54)	0.321	–	**–**
Acceptance of assisted reproductive technology → Practice	−1.87 (−2.65, −1.08)	**< 0.001**	−1.23 (−1.94, −0.53)	**0.001**	−0.63 (−1.02, −0.24)	**0.001**
Acceptance of painless egg retrieval surgery → Practice	−1.87 (−2.65, −1.08)	**< 0.001**	−1.03 (−1.74, −0.32)	**0.005**	−0.83 (−1.23, −0.43)	**< 0.001**

## Discussion

Our study revealed that infertile women in the Northwest Region displayed moderate levels of KAP concerning ART and painless egg retrieval. Significant positive correlations existed among the KAP scores, with knowledge indirectly influencing practice. Education level and experience with assisted reproductive technology and painless egg retrieval were key factors affecting KAP scores.

Aligning with our results, only 12.7% of Australian women seeking fertility assistance could accurately identify the fertile window of their menstrual cycle (16). Comparably, although women in the Northwest Region had a basic understanding of ART, many were unaware of more nuanced details, such as treatment procedures and side effects. Contrasting with our results, a study from Iran indicated that the infertile couples held predominantly negative attitudes toward ART (17). Also, another study reported that women undergoing IVF-ET procedures had inadequate knowledge and negative attitudes toward embryo transfer (11). Healthcare providers should enhance counseling with visual aids and digital platforms for better accessibility. Besides, peer support groups are encouraged to inform decision-making and alleviate concerns. The relatively high attitude scores in our study may be attributed to increasing social acceptance of ART in the sampling region and a growing awareness of its benefits. As for practices, only limited proportion of infertile women in our study actually pursued ART treatments and related information. The low uptake could be explained by several factors, including financial constraints, geographic disparities in access to healthcare facilities offering ART, and cultural hesitancy (18). Policymakers should also consider subsidizing ART services and expanding insurance coverage to reduce financial barriers, ultimately promoting equitable access to fertility treatments.

In the knowledge dimension, the high familiarity was with the primary goal of ART and the definition of painless egg retrieval. The findings suggest that the participants had basic understandings of ART, which aligned with previous evidence of women undergoing ART (19). However, the substantial lack of familiarity with the risks of painless egg retrieval underscored a critical gap in the education of infertile women. Ovarian hyperstimulation syndrome arises from ovarian stimulation during ART procedures, which be life-threatening in severe cases (20). Also, ovarian infection can lead to pelvic infections, abdominal pain, and even long-term fertility issues (21). The low level of awareness can be attributed to the focus of healthcare professionals on treatment success rather than on educating patients about risks. Besides, only 19.60% were familiar with the anatomical cause of pain during egg retrieval. Since ART clinics typically prioritize medical explanations over patient-centered education, patients can get insufficient preparation for the physical sensations during egg retrieval (22). Educating women about the side effects and risks of painless egg retrieval, as well as the physiological basis of pain during the procedure, could improve the overall patient experience.

In the attitude dimension, the highest agreement was with the reliability of ART in helping infertile patients achieve reproductive goals. Consistently, the success rates of ART have significantly improved over the years, bolstering its reputation as a viable solution to infertility (23). Conversely, the least proportion held positive attitudes toward the negligible side effects of painless egg retrieval. Consistently, a survey-based study from the United States reported medium or high levels of ART-related stress and depression among infertile individuals (24). Besides, due to the use of sedation and anesthesia in painless egg retrieval, the lack of understanding or inadequate communication can lead to persistent concerns (25). Moreover, less than half of participants expressed a positive attitude toward ART not conflicting with family planning and eugenics policies. The hesitancy in accepting ART could be related to concerns about its ethical implications, potential for “designer babies”, or fear of unintended societal consequences (26). In light of these concerns, patient education must not only focus on the medical and procedural aspects of ART, but also provide a comprehensive understanding of the legal, cultural, and ethical dimensions of ART. Ensuring that women feel supported in navigating these issues will be essential for improving their attitudes toward ART.

Most participants expressed the willingness to try ART, particularly IVF, to achieve reproductive goals. In line with our findings, about 13% of US women of reproductive age seek infertility services, with ART being a common treatment choice (27). This growing willingness can be attributed to advancements in ART technology, improved clinical outcomes, and the broader social acceptance of these procedures. In contrast, the lowest adherence was observed for communicating and consulting with a doctor or professional about ART. Similarly, only limited proportion of participants actively sought more information about ART and painless egg retrieval. One possible explanation was that many women may feel overwhelmed by the medical aspects of ART, leading them to avoid consultations and information-seeking behaviors. Previous research has highlighted that the emotional and psychological burden of infertility can discourage women from actively seeking information or professional advice (28). Additionally, barriers such as insufficient time, financial constraints, and a lack of accessible healthcare resources may play a role in limiting consultations. Clinicians should ensure that their consultations are patient-centered, transparent, and inclusive of comprehensive discussions about ART procedures, potential risks, and the emotional aspects of treatment. Furthermore, providing women with easily accessible resources, such as pamphlets and online content, could help increase their engagement and understanding of ART. Beyond these practical barriers, sociocultural factors may also contribute to the low levels of communication with doctors and limited proactive learning about ART. In China, reproductive health topics are often regarded as private or sensitive, which can discourage open dialogue with healthcare providers. Moreover, traditional family structures may place decision-making power with spouses or older adult, further reducing women's initiative to seek information independently. In comparison, studies in Western countries, such as the United States and Australia, report higher rates of proactive information-seeking and patient–doctor communication, partly due to greater emphasis on patient autonomy and readily available digital health resources (16, 27). These cross-cultural contrasts suggest that interventions in China must not only address financial and logistical challenges, but also consider cultural norms and family dynamics when designing patient education strategies.

In alignment with the health belief theory, positive and direct relationships were identified among KAP scores (29). In other words, individuals with higher levels of knowledge may be more likely to view ART and painless egg retrieval positively, increasing their willingness to pursue these treatments. Besides, knowledge and education level had an indirect association with practice. The results suggest that knowledge serves as a foundational element that can influence psychological and behavioral factors, leading to altered practices. Several influential factors of KAP were reported. First, the negative influence of a lack of acceptance toward ART and painless egg retrieval on practice was found. This highlighted the importance of addressing misconceptions about the treatments to increase patient readiness. Second, women with higher education levels had better knowledge and attitude scores, possibly due to elevated health literacy and socioeconomic status (30).

This study had several limitations. The cross-sectional design restricted causal inferences between KAP and baseline factors. Additionally, the small sample size and regional sampling may limit generalizability of our findings. Self-reported data may also introduce social desirability bias, potentially inflating KAP scores (31). Moreover, as the survey was distributed via the WeChat-based Wenjuanxing platform, participants may have been skewed toward those who are more digitally literate and predominantly urban residents, potentially limiting representativeness of the findings.

In China, national conditions such as the high financial burden of ART, limited health insurance coverage, and regional disparities in ART service availability also affect women's knowledge, attitudes, and practices. These factors may partly explain the moderate KAP levels observed in this study.

## Conclusion

In conclusion, infertile women in the Northwest Region of China exhibited moderate KAP regarding ART and painless egg retrieval. Positive and direct correlations were found among KAP scores, with knowledge indirectly influencing practice. Targeted educational interventions on doctor-patient communication and peer support should be implemented, particularly for women with lower education levels and limited ART experience.

## Data Availability

The original contributions presented in the study are included in the article/Supplementary material, further inquiries can be directed to the corresponding author.
