# Supplementary material for: Knowledge, attitudes, and practices toward assisted reproductive technology and painless egg retrieval among infertile women in the northwest region of China
Source: Front Public Health. 2025 Oct 2;13:1614206. doi: 10.3389/fpubh.2025.1614206 (PMC12528081; doi:10.3389/fpubh.2025.1614206)
Supplement: Supplementary file 1 [file Image_1.pdf]

Supplementary File 1. Validation and Structural Equation Model Results of the KAP Questionnaire

CFA:

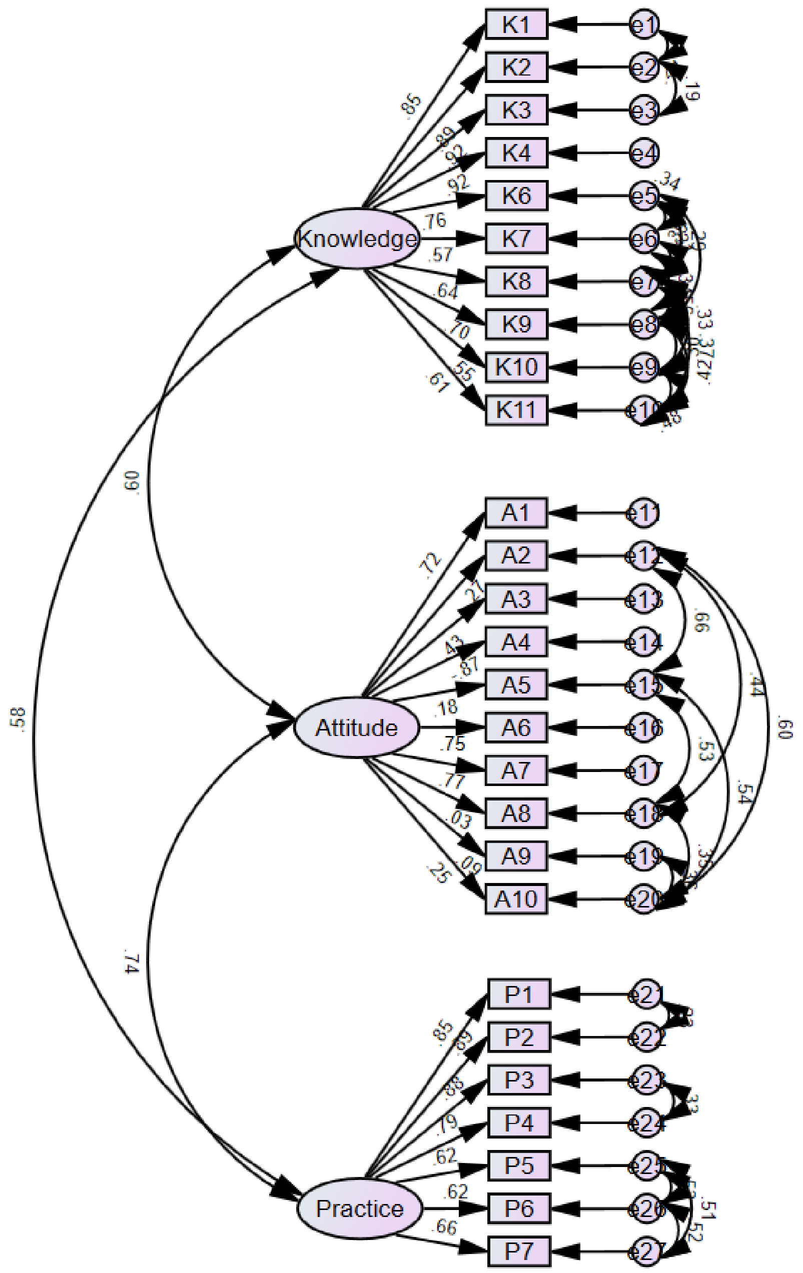

Note: Ovals represent latent variables (Knowledge, Attitude, and Practice), rectangles represent observed items (K1–K11, A1–A10, P1–P7), and circles represent error terms. Standardized factor loadings are shown on the arrows from latent variables to observed items. Single-headed arrows between latent variables indicate standardized path coefficients.

Indicators	Reference	Actual
CMIN/DF	1-3: Excellent, 3-5: Good	2.436
RMSEA	<0.08: Good	0.060
IFI	>0.8: Good	0.947
TLI	>0.8: Good	0.937
CFI	>0.8: Good	0.947

			**Estimate**	**Standardized Estimate**	**S.E.**	**C.R.**	**P**
K1	<---	Knowledge	1	0.847			
K2	<---	Knowledge	1.007	0.885	0.033	30.066	***
K3	<---	Knowledge	1.069	0.919	0.043	24.948	***
K4	<---	Knowledge	1.091	0.918	0.044	24.961	***
K6	<---	Knowledge	0.968	0.758	0.053	18.141	***
K7	<---	Knowledge	0.77	0.568	0.063	12.272	***
K8	<---	Knowledge	0.879	0.64	0.061	14.299	***
K9	<---	Knowledge	0.915	0.705	0.056	16.299	***
K10	<---	Knowledge	0.778	0.554	0.065	11.924	***
K11	<---	Knowledge	0.829	0.612	0.061	13.509	***

			Estimate	Standardized Estimate	S.E.	C.R.	P
A1	<---	Attitude	1	0.717			
A2	<---	Attitude	0.61	0.267	0.121	5.062	***
A3	<---	Attitude	0.787	0.435	0.096	8.232	***
A4	<---	Attitude	-1.265	-0.866	0.079	-16.099	***
A5	<---	Attitude	0.348	0.176	0.104	3.335	***
A6	<---	Attitude	1.098	0.749	0.078	14.114	***
A7	<---	Attitude	1.17	0.772	0.08	14.54	***
A8	<---	Attitude	0.055	0.03	0.099	0.559	0.576
A9	<---	Attitude	0.226	0.087	0.137	1.655	0.098
A10	<---	Attitude	0.501	0.25	0.106	4.727	***
P1	<---	Practice	1	0.849			
P2	<---	Practice	1.019	0.894	0.039	26.429	***
P3	<---	Practice	1.009	0.878	0.049	20.785	***
P4	<---	Practice	0.92	0.79	0.052	17.753	***
P5	<---	Practice	0.972	0.617	0.074	13.212	***
P6	<---	Practice	1.012	0.623	0.076	13.357	***
P7	<---	Practice	1.095	0.66	0.076	14.369	***

KMO=0.931 (P<0.001)
