# Supplementary material for: Knowledge, attitudes, and practices toward assisted reproductive technology and painless egg retrieval among infertile women in the northwest region of China
Source: Front Public Health. 2025 Oct 2;13:1614206. doi: 10.3389/fpubh.2025.1614206 (PMC12528081; doi:10.3389/fpubh.2025.1614206)
Supplement: Supplementary file 2 [file Table_1.docx]

**Table S1. Spearman correlation analysis of KAP scores**

|  | **Knowledge** | **Attitude** | **Practice** |
| --- | --- | --- | --- |
| **Knowledge** | 1 |  |  |
| **Attitude** | 0.246 (P<0.001) | 1 |  |
| **Practice** | 0.589 (P<0.001) | 0.301 (P<0.001) | 1 |

**Table S2. Model fit indices of structural equation model**

| Indicators | Reference | Results |
| --- | --- | --- |
| RMSEA | <0.08 Good | <0.001 |
| SRMR | <0.08 Good | 0.009 |
| TLI | >0.80 Good | 1.025 |
| CFI | >0.80 Good | 1.000 |

Abbreviations: RMSEA, Root Mean Square Error of Approximation; SRMR, Standardized Root Mean Square Residual; TLI, Tucker-Lewis Index; CFI, Comparative Fit Index
